# The Whip and Spur

**Published:** 1884-11

**Authors:** 


### THE WHIP AND SPUR.

THAT man or woman who, when feeling too weak to perform the allotted task, flies to some brain stimulant to secure temporary strength, is certainly a very thoughtless person, if not almost a suicide. It is this practice of seeking power from wine or brandy, or whiskey or tobacco, or coffee, or tea, or opium, which brings sudden death to so many gifted men. It is this habit—the habit of working on stimulants— which makes a multitude of drunkards. The best possible thing for a man to do when he feels too tired to perform a task, or too weak to carry it through, is to go to bed and sleep a week if he can; this is the only true recuperation of brain-power; the only actual renewal of brain forces, because during sleep the brain is, in a sense, at rest, in a condition to receive and appropriate particles of nutriment from the blood which take the place of those which have been consumed in previous labor. Mere stimulants supply nothing; they only goad the brain, force it to a greater consumption of its substance, until that substance has been so fully exhausted that there is not power enough left to receive a supply; just as men are sometimes so near death by thirst and starvation that there is not strength enough left to swallow anything, and all is over. The true way is to rest the brain when it is exhausted; and renewed force comes to it in no way so surely and so rapidly as in early and abundant sleep. To so inflame the brain by overwork as to deprive it of the power of repose and recuperation, is almost certain death, and is absolutely certain and permanent injury to body and brain.

